# The Importance of Histopathological Examination to the Final Diagnosis of Peripheral Odontogenic Tumors: A Case Report of a Peripheral Odontoma

**DOI:** 10.1155/2019/9712816

**Published:** 2019-09-08

**Authors:** Marcio Augusto de Oliveira, Bruna Reis, Debora Pallos, Yeon Jung Kim, Paulo Henrique Braz-Silva, Fabiana Martins

**Affiliations:** ^1^Department of Stomatology, School of Dentistry, University of São Paulo, Av Prof. Lineu Prestes 2227, 05508-000 São Paulo, Brazil; ^2^Dental School, University of Santo Amaro, R. Prof. Eneas de Siqueia Neto 340, 04829-300 São Paulo, Brazil; ^3^Laboratory of Virology, Institute of Tropical Medicine of São Paulo, University of São Paulo, v. Dr. Enéas Carvalho de Aguiar 470, 05403-000 São Paulo, Brazil

## Abstract

A 30-year-old Caucasian man presented with an 18-month history of an asymptomatic calcified mass, located on the buccal side of the alveolar ridge. Medical records did not present any underlying conditions. On intraoral examination, the lesion was located on the right side of the maxilla, showing mucosal fenestration with mineralized tissue measuring approximately 1 cm in diameter. Radiographic examination showed multiple radiopaque masses. Incisional biopsy was performed, and histological analysis revealed a presence of enamel matrix, dentin, and cementum, resembling tooth-like structures. Surgical removal was offered after the diagnostic confirmation of peripheral odontoma, but the patient refused because of the asymptomatic nature of the lesion.

## 1. Introduction

Odontomas are the most common type of odontogenic tumors, being considered hamartomas when associated with dental development failure [1]. Two types of odontomas can be recognized: complex and compound odontomas, with the former being characterized by a mineralized mass and the latter by multiple small calcifications [2, 3]. Clinically, odontomas can be intraosseous or extraosseous. The intraosseous type can eventually erupt into the oral cavity, whereas the peripheral odontoma (PO) occurs in the soft tissues and is considered rare, with a higher tendency to exfoliate [4].

PO is extremely uncommon, with less than 25 cases reported in the literature [4, 5]. PO arises in young individuals and children, rarely reported in adults. Histologically, both complex and compound types can be seen in more than 50% of the cases affecting the anterior region of the maxilla [4].

The purpose of this article is to present a new case of erupted peripheral odontoma of the maxilla in a 30-year-old patient and to review and discuss the characteristics of the cases of PO in the craniofacial region described in the literature.

## 2. Case Report

**Figure 1:**
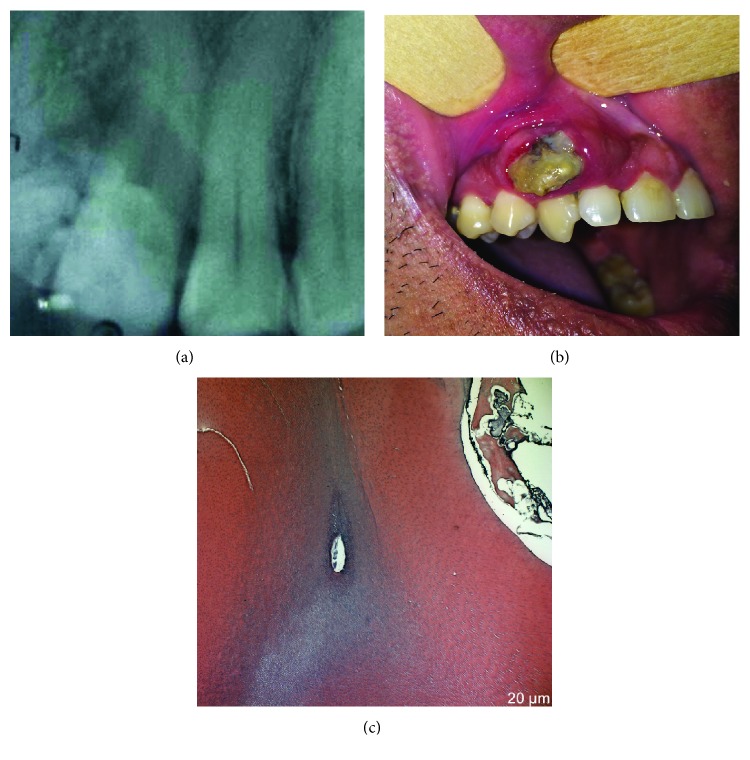
Radiographic examination showing multiple radiopaque masses (a). Fenestration of the mucosa with a display of mineralized tissue (b). Histological analysis revealed the presence of enamel matrix, dentin, and cementum, resembling tooth-like structures (c).

A 30-year-old male patient was referred with a diagnosis of an asymptomatic calcified mass located on the right side of the anterior maxilla, lasting 18 months. On the intraoral examination, a mucosal cutaneous fenestration with a mineralized tissue measuring approximately 1 cm in diameter was observed (Figure 1(a)). A periapical radiograph revealed an image exhibiting discrete radiopacity in the region of upper premolars (Figure 1(b)). The diagnostic hypothesis was maxillary exostosis.

Incisional biopsy was performed for the removal of the fragment, which then was placed in 10% neutral-buffered formalin and sent for histopathological analysis. The gross examination of the calcified specimen revealed a yellowish tumor with 4 × 4 × 5 mm.

The histopathological analysis revealed structures composed of enamel, dentin, pulp chamber, and cement in the same order of arrangement as that of a normal tooth. A mature tubular dentin and an enamel matrix were also observed (Figure 1(c)).

After the diagnostic confirmation of erupted peripheral odontoma, surgical removal was performed to reduce the lesion. The patient was free of symptoms after the procedure, and no complications were recorded.

## 3. Discussion

Odontomas, by definition, refer to any tumor of odontogenic origin, although these entities are truly considered hamartomas [2]. Odontomas occur at any age but are most commonly seen in the first two decades of life [2], which is not coincident with the present case of a 30-year-old adult, denoting the rarity of this lesion.  Table 1 demonstrates a review of peripheral odontomas in adults and children, reported in English literature (Table 1 [3, 4, 6–15]). Among the cases described, only 5 [2, 9, 11, 13, 15] were histologically confirmed cases of erupted peripheral compound odontoma in adults, and among these, the present case and three additional cases were not associated with an impacted tooth [9, 13, 15–22].

PO is usually asymptomatic and detected during routine radiographic examinations or once there is a delay in tooth eruption [Rajendran et al., 2012]. The differential diagnosis of OP includes other tumors of odontogenic origin, exostoses, and osteomas [6], with the latter being considered our first diagnostic hypothesis.

The histogenesis of odontomas is primarily associated with remnants of soft tissues of the odontogenic epithelium, such as the gingival rests of Serres, which could lead to the production of mineralized structures similar to teeth as a result of odontoblastic hyperactivity and changes in the genetic component responsible for controlling dental development, including a reduction of epithelial-mesenchymal interactions [2].

This condition can also be attributed to some pathological conditions, such as local trauma, inflammatory process, and infectious and genetic anomalies [4, 5].

Some of the peripheral odontomas reported in the literature might be erupted odontomas because of intraosseous lesions, often related to unerupted or spontaneously erupted teeth [2]. However, it can be postulated that the eruptive force of nonerupted teeth plays an important role in the eruption of odontoma. In the absence of unerupted teeth, some authors assume that the odontoma eruption is caused by local bone resorption, which may involve both the bone remodeling of the jaws and the increase in the size of the tumor over time, since movement forces are not linked to fibroblast contractility, unlike teeth [7]. Another hypothesis described in the literature is that PO is an erupted form of an extraosseous mesiodens [1].

In the present case, the patient was referred to an evaluation due to exposure of the mineralized tissue in the oral cavity, suggesting a growth of the lesion and the presence of mild discomfort in the adjacent periodontal area. The type of force that may have led to mucosal fenestration was probably caused by physiological bone resorption, since there was no report of delayed dental eruption.

Histologically, PO resembles intraosseous odontomas, which can be classified as compound and complex. However, the absence of bone tissue is a finding also observed in the present case. This fact occurs due to the absence of bone erosion under the tumor, supporting the hypothesis of the development of this odontoma in the gingival tissue [6, 8].

Clinically, some authors have described odontomas that erupted in older patients and whose lesions were histologically characterized as complex odontomas associated with noneruption of posterior teeth [6]. Conversely, the lesion described was located in gingival tissues and was not associated with an impacted tooth, being histologically described as a compound odontoma.

Peripheral odontoma is a rare benign odontogenic lesion that can be treated by local excision with good results. Both young and adult patients can present these alterations. A comprehensive evaluation with radiographic and histological examinations is important to establish the differential diagnosis and prevent unnecessary extensive resections.

## Figures and Tables

**Table 1:** Review of peripheral odontoma cases described in the literature.

Author	Age^∗^	Gender	Location	Erupted PO	Histopathologic diagnosis
Present case	30	Male	Anterior maxillary region	Yes	Compound odontoma
Custódio et al.	11	Female	Anterior maxillary region	No	Complex odontoma
Sfakianou et al.	7 Mo^∗∗^	Male	Posterior mandible region	No	Peripheral developing odontoma
Ahmed	24	Male	Posterior mandible region	Yes	Complex odontoma
Bagewadi et al.	22	Male	Posterior mandible region	Yes	Complex odontoma
Bereket et al.	19	Male	Posterior maxillary region	Yes	Compound odontoma
Kudva et al.	23	Male	Posterior mandible region	Yes	Complex odontoma
Raval et al.	22	Male	Anterior maxillary region	Yes	Compound odontoma
Ohtawa et al.	10	Female	Posterior maxillary region	Yes	Complex odontoma
Arunkumar et al.	22	Male	Posterior maxillary region	Yes	Complex odontoma
Tejasvi and Babu	22	Female	Anterior mandible region	Yes	Compound odontoma
Friedrich et al.	3	Male	Posterior maxillary region	No	Peripheral developing odontoma
Serra-Serra et al.	11 27	Male Male	Posterior maxillary region Anterior mandible region	Yes Yes	Complex odontoma Compound odontoma
Shekar et al.	15	Female	Posterior mandible region	Yes	Compound odontoma
Silva et al.	5 Mo 8 Mo	Male Male	Anterior maxillary region Anterior maxillary region	No No	Peripheral developing odontoma Peripheral developing odontoma
Ilief-Ala et al.	2	Female	Posterior maxillary region	Yes	Complex odontoma
Vengal et al.	23	Male	Posterior mandible region	Yes	Complex odontoma
Junquera et al.	23	Female	Posterior maxillary region	Yes	Complex odontoma
Ide et al.	39	Male	Anterior maxillary region	No	Complex odontoma
Ledesma-Montes et al.	3	Female	Posterior mandible region	No	Compound odontoma
Giunta et al.	21	Male	Posterior mandible region	No	Compound odontoma

^∗^Patient's age at the time of the oral examination. ^∗∗^Months
